# Bile Bacterial Colonization Increases Risk of Postoperative Pancreatic Fistula and Worsens Overall Survival Following Pancreatoduodenectomy

**DOI:** 10.3390/jcm15041566

**Published:** 2026-02-16

**Authors:** Natalia Olszewska, Tomasz Guzel, Kaja Śmigielska, Piotr Paluszkiewicz, Agnieszka Milner, Edyta Podsiadły, Maciej Słodkowski

**Affiliations:** 1 Department of General, Gastroenterological and Oncological Surgery, Medical University of Warsaw, 02-097 Warsaw, Poland; smigielskka@gmail.com (K.Ś.); piotr_paluszkiewicz@o2.pl (P.P.); maciej.slodkowski@wum.edu.pl (M.S.); 2 Microbiological Laboratory, University Center for Laboratory Medicine, Medical University of Warsaw, 02-097 Warsaw, Poland; agnieszka.milner@uckwum.pl; 3 Department of Dental Microbiology, Medical University of Warsaw, 02-097 Warsaw, Poland; edyta.podsiadly@uckwum.pl

**Keywords:** pancreatoduodenectomy, bile bacterial colonization, pancreatic cancer, survival, postoperative pancreatic fistula

## Abstract

**Background:** Postoperative pancreatic fistula (POPF) is a major source of morbidity following a pancreatoduodenectomy (PD), often delaying or precluding adjuvant chemotherapy and potentially compromising long-term oncologic outcomes. While established risk models focus on anatomical and biochemical factors, the role of biliary microbiota remains underexplored. This study aimed to assess relationship between bacteriobilia and the incidence of POPF, as well as its impact on overall survival (OS) in patients undergoing a PD for pancreatic ductal adenocarcinoma (PDAC). **Methods:** We analyzed the medical histories of 725 patients with a pancreatic tumor who were qualified for surgery between 2017 and 2022. This retrospective cohort study included 138 patients who underwent a PD for histologically confirmed PDAC. Intraoperative bile cultures were obtained and analyzed for microbial presence and resistance patterns. **Results:** Bacteriobilia was detected in 76.8% of patients, including bacteria with resistance mechanisms (BRM) present in 12.3% of bile samples. Bacterial bile colonization conferred an increased odds of POPF grade B (OR 5.11; *p* = 0.088), whereas BRM were strongly predisposed to POPF grade C (OR 4.97; *p* = 0.026). Upon a multivariate analysis, bacteriobilia independently drove clinically relevant POPF and POPF grade B (OR 5.50; *p* = 0.034 and OR 8.04; *p* = 0.048, respectively), while BRM remained a key determinant of POPF grade C (OR 6.17; *p* = 0.047). Beyond morbidity, bile colonization markedly impaired overall survival irrespective of tumor stage (26.7 vs. 54.7 months; log-rank *p* = 0.009). **Conclusions:** Bacterial bile colonization may contribute not only to higher rates of POPF but to a significantly reduced OS in patients undergoing a PD for PDAC. Bacteriobilia should be considered as a prognostic factor for worse survival after a PD.

## Simple Summary

We analyzed bile samples collected during pancreatoduodenectomy in patients with PDAC. The importance of this study lies in the conclusion that bacteriobilia might be associated with higher rates of postoperative pancreatic fistula and shorter overall survival, independently of the cancer stage, and thus should be recognized as a negative prognostic factor following PD.

## 1. Introduction

Pancreatic ductal adenocarcinoma (PDAC) remains one of the most lethal malignancies of the gastrointestinal tract, with a 5-year survival rate ranging from 6% to 12%, depending on the stage at diagnosis and the treatment modalities applied [1]. Due to its aggressive biological behavior and because it frequently presents with late-onset symptoms, curative treatment is achievable only for a minority of patients. The cornerstone of therapy is radical surgical resection combined with regional lymphadenectomy, followed by adjuvant chemotherapy [2].

A pancreatoduodenectomy (PD) is the standard surgical approach for tumors localized in the head of the pancreas. Despite advances in surgical techniques and perioperative care, a PD remains one of the most complex procedures in abdominal surgery, associated with a high morbidity rate ranging from 30% to 55%, depending largely on the surgeon’s experience and institutional volume [3,4,5,6]. Among the postoperative complications, anastomosis leakage leading to postoperative pancreatic fistula (POPF) remains the most feared and clinically significant. Clinically relevant POPF, defined by the International Study Group on Pancreatic Fistula (ISGPS) as grade B or C, occurs in approximately 7% to 33% of cases and is associated with a mortality rate of up to 25% in POPF grade C [3,4,5,6,7]. Clinically significant postoperative complications, particularly POPF, are known to compromise oncological outcomes. Their occurrence frequently leads to delays in the initiation of adjuvant chemotherapy and, in some cases, may result in patients being unable to receive systemic treatment entirely [8,9].

Despite various efforts to optimize surgical techniques, perioperative management, and anastomotic reconstruction strategies, the exact pathophysiological mechanisms underlying the development of POPF remain incompletely understood [10,11]. Recently, attention has turned toward the potential role of microbial factors in the pathogenesis of postoperative complications. Emerging evidence suggests that bacterial colonization of the biliary tract may contribute to impaired healing of the pancreatico-enteric anastomosis and increase the risk of POPF, as well as other infectious complications, thereby influencing both short-term outcomes and long-term survival [12,13,14].

The aim of this study is to evaluate the impact of biliary bacterial colonization on the incidence of postoperative complications—particularly pancreatic fistula—following a PD for PDAC, and to explore its potential association with long-term oncological outcomes.

## 2. Materials and Methods

We conducted a retrospective study including 725 patients who were qualified for surgical treatment due to a pancreatic tumor at the Department of General Gastroenterological and Oncological Surgery, University Clinical Center of the Medical University of Warsaw, between 2017 and 2022. This institution is recognized as a high-volume center [15]. Demographic characteristics, laboratory parameters, and complete medical histories of the study population were retrospectively reviewed and analyzed.

This study was carried out in accordance with the principles of the Declaration of Helsinki and received approval from the institutional Bioethics Committee. The reporting of the study findings adheres to the STROBE (Strengthening the Reporting of Observational Studies in Epidemiology) guidelines.

### 2.1. Study Design and Eligibility Criteria

To ensure the standardization and homogeneity of the study cohort, clearly defined inclusion and exclusion criteria were applied. Of the initial 725 patients evaluated, only those who underwent a pancreatoduodenectomy (*n* = 314) for a tumor located in the head of the pancreas were considered eligible for inclusion. Patients who had undergone other types of pancreatic procedures—such as distal pancreatectomy, total pancreatectomy, local tumor excision, or exploratory laparotomy without resection—were excluded from further evaluation. Patients who had received preoperative chemotherapy were also excluded from the analysis to avoid any potential bias related to treatment response or altered tissue characteristics.

**Figure 1:**
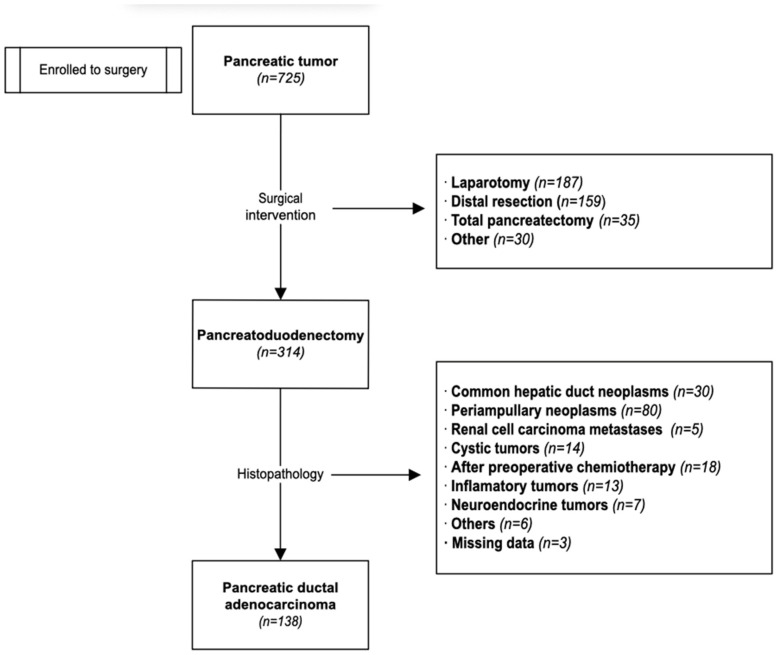
Flowchart illustrating the study design and the patient selection process.

Following histopathological verification, we conducted a retrospective cohort study. For the statistical analysis, we enrolled only those patients with a confirmed diagnosis of PDAC (*n* = 138) (Figure 1).

### 2.2. Surgical Technique

Pancreatoduodenectomies were performed by three highly experienced pancreatic surgeons. Use of the classical Whipple procedure or the pylorus-preserving Traverso–Longmire technique was determined by tumor location and intraoperative assessment. Reconstruction of the remaining pancreas was carried out using pancreatico-enteric anastomosis, either by the duct-to-mucosa or the invagination technique. The choice of anastomotic method was guided by the surgeon’s expertise and the anatomical characteristics of the pancreatic duct and surrounding tissues.

Biliary reconstruction was performed by creating a hepaticojejunostomy using either a continuous single-layer suture or interrupted single sutures, depending on the diameter and condition of the common hepatic duct. Gastrointestinal continuity was restored using one of two configurations: a gastrojejunostomy with an omega loop and Braun anastomosis, or a duodenojejunostomy with an omega loop, based on the resection technique and intraoperative findings.

In most cases an oncological lymphadenectomy was carried out following the standardized Heidelberg technique, which involves an en-bloc dissection of the lymphatic tissue within the anatomical triangle formed by the celiac axis/hepatic artery, the superior mesenteric artery, and the portal vein/superior mesenteric vein [16,17]. This approach ensures comprehensive clearance of regional lymph nodes and is considered a key element of radical pancreatic head resection [18]. An antibiotic prophylaxis of 2 g of Cefazoline and 500 mg of Metronidazol were administered within 30 min before incision, with intraoperative redosing every 4 h until wound closure.

Patients in whom intraoperative exploration revealed distant metastases or locally advanced, non-resectable tumors were not subjected to pancreatic resection, in accordance with oncological safety principles and current surgical guidelines [19]. Postoperative Pancreatic Fistula (POPF) was defined and classified according to the criteria established by the ISGPS [20].

### 2.3. Microbiological Identification Methods

Intraoperative bile samples for microbiological culture were obtained immediately after transection of the common bile duct, using a new scalpel blade to ensure aseptic conditions. Samples were cultured using bacteriological media according to laboratory procedures. Bacterial cultures were performed on Columbia Blood Agar with 5% sheep blood, MacConkey Agar, Schaedler Agar with 5% sheep blood, Schaedler Agar supplemented with colistin–nalidixic acid (CNA), and Schaedler Agar supplemented with kanamycin and vancomycin. Bacterial identification was performed with MALDI-TOF mass spectrometry with Microflex LT mass (Bruker Daltonics, Bremen, Germany) using the MBT Compass IVD software (Bruker Daltonics, Bremen, Germany) according to the manufacturer’s instructions. Bacteria classified as drug-resistant with resistance mechanisms (BRM) were defined as isolates exhibiting specific, well-characterized antimicrobial resistance mechanisms conferring reduced susceptibility or resistance to antimicrobial agents; these isolates were grouped and analyzed as a separate subgroup. Production carbapenemases (MBL, KPC OXA-48) by isolates was investigated by DDST-EDTA (double-disk synergy test with ethylenediaminetetraacetic acid) and detected MBL, and a CDT (combined disk test) detected KPC. Production of OXA-48 was detected using an antibiogram disc with temocillin. The Rapidec^®^ Carba NP and/or the GeneXpert qualitative real-time PCR method (Cepheid, Sunnyvale, CA, USA) were also performed. ESBL was detected with the phenotypic confirmation method—double-disk synergy test (DDST).

### 2.4. Statistical Analysis

A statistical analysis was performed using IBM SPSS Statistics (v.30.0.0.0). Descriptive data are presented as means with standard deviations or as frequencies and percentages. Group comparisons used the Chi-squared or Fisher’s exact test for categorical variables, and the Student’s *t*-test or the Mann–Whitney U test for continuous variables, depending on the distribution. For ordinal outcomes, linear-by-linear association tests assessed the trends. Associations with POPF were evaluated using the univariate odds ratios (OR) with 95% confidence intervals. A multivariable analysis was performed to identify independent risk factors for postoperative pancreatic fistula (POPF). P-values in the multivariable logistic regression were calculated using the likelihood ratio test, while confidence intervals were estimated using the Wald method. Overall survival was analyzed with Kaplan–Meier curves and log-rank tests. Surgical technics were not considered under analysis in this study.

To evaluate the impact of bacterial colonization (positive bile culture) and the stage of pancreatic cancer on OS, a survival analysis was performed using the Cox proportional hazards regression model. Nodal status (N stage) was used as a surrogate marker of cancer advancement. For the purpose of this analysis, patients were categorized into two prognostic groups based on the extent of lymph node involvement: those with N0 were classified as having low-stage cancer, while those with N1 or higher were considered to have high-stage cancer. This binary classification was applied to enable an efficient stratification of outcomes and an interaction analysis with bacterial colonization status. A multivariable model was constructed including both variables and an interaction term (culture × N stage) to assess whether the prognostic effect of bacterial colonization depended on cancer advancement. A *p*-value < 0.05 was considered statistically significant. The proportional hazards assumption was formally tested and met for all Cox regression models.

## 3. Results

### 3.1. Baseline Characteristics of the Study Population

Characteristics of the study cohort, which comprised 138 patients who underwent a pancreatoduodenectomy, are presented in Table 1. Most patients were classified as ASA physical status II, reflecting moderate systemic comorbidity.

### 3.2. Bile Colonization and Postoperative Pancreatic Fistula

Positive bile cultures were found in 76.8% of the patients, with BRM isolated in 12.3% of the cases. Clinically relevant POPF were identified in 24 patients (17.4%), including 16 grade B and 8 grade C fistulas (11.6%, and 5.8%, respectively) (Table 1).

**Figure 2:**
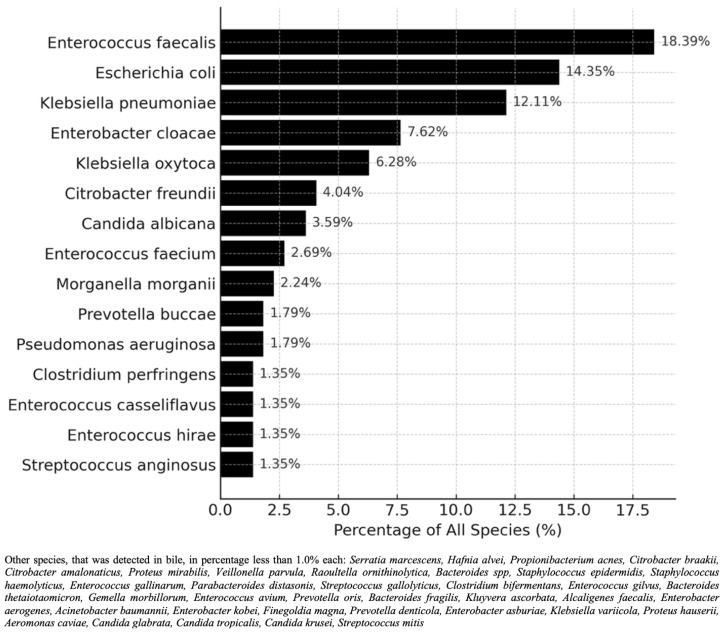
Microbiological spectrum of bile cultures in the study cohort.

The most frequently isolated microorganisms from the bile cultures are presented in Figure 2. Percentages indicate the proportion of each microorganism among all isolates identified in the bile samples. Species detected in fewer than 1.0% of cultures are not displayed individually and are summarized below the figure.

Bacterial colonization of bile was polyetiological (223 bacterial isolates consisting of 53 different bacterial species), with a mean of three different microbial species isolated per sample.

In 17 bile samples, bacteria with resistance mechanisms (BRM) were identified, representing 16.04% of all patients with positive bile cultures (17 patients with BRM in 106 of all bile positive patients). A total of 23 BRM isolates were cultured from these patients, with some individuals harboring two different BRM species. The most frequent resistance mechanism was extended-spectrum β-lactamase (ESBL) production, detected in 17 of 23 isolates (73.9%), vancomycin-resistant enterococcus (VRE) accounted for 13.0% of the isolates, while NDM-producing strains were identified in three patients (Table 2).

### 3.3. Association Between Bile Bacterial Colonization and Postoperative Pancreatic Fistula

Bacteriobilia showed a higher rate of POPF grade B (14.2% vs. 3.1%), with a large effect size, although the estimate was imprecise (OR 5.11, *p* = 0.088). Grade C fistulas developed in 4.7% vs. 9.4% of patients, with no significant relationship to bile contamination (*p* = 0.323). In the analyzed cohort, the presence of BRM was not associated with the occurrence of POPF grade B. For POPF grade C, patients colonized with BRM had a higher, statistically significant risk (OR = 4.97, *p* = 0.026) (Table 3).

Multivariable logistic regression was performed to assess whether positive microbiological culture in bile (bacteriobilia) and BRM are independent risk factors for occurrence of POPF. Models were adjusted for age, sex (male), BMI, ASA physical status, and pancreatic duct anastomosis technique (duct to mucosa vs. invagination). Three endpoints were analyzed: POPF grade B, POPF grade C, and clinically relevant POPF (grade B + grade C). After adjustment for patient-related factors and anastomotic technique, bacteriobilia was independently associated with the clinically relevant POPF (grades B/C) and POPF grade B (OR 5.50, *p* = 0.034 and OR 8.04; *p* = 0.048, respectively), whereas BRM were significant independent predictors of POPF grade C (OR 6.17, *p* = 0.047) (Table 4). However, the confidence interval remained wide, reflecting limited precision due to the low number of events. There were no statistically significant results for other confounders (age, sex, BMI, ASA, anastomosis technique).

### 3.4. Bile Colonization and Overall Survival After a PD

**Figure 3:**
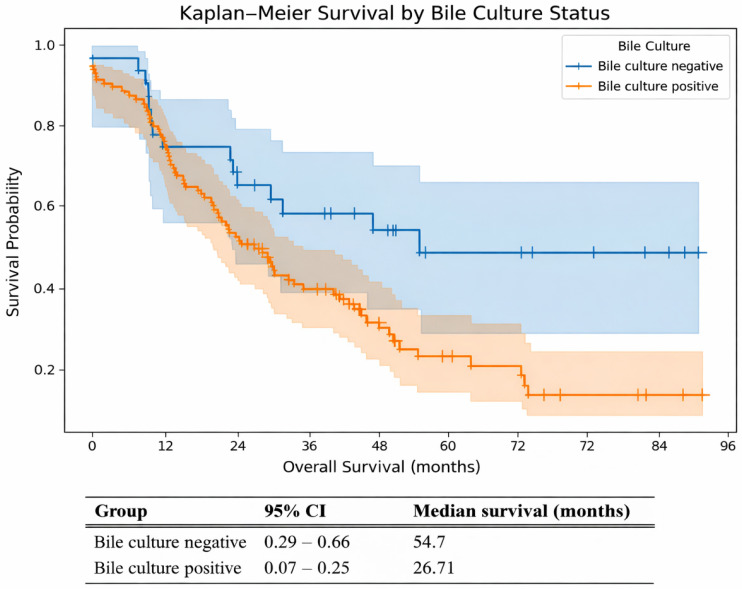
Kaplan–Meier overall survival curves stratified by intraoperative bile culture status. Shaded areas indicate 95% confidence intervals, and tick marks represent censored observations. Median overall survival values are provided below the figure.

Patients with positive bile cultures (orange line) had a significantly reduced overall survival (OS) compared to those with negative cultures (blue line) (log-rank test, *p* = 0.009). Median survival was markedly shorter in the bile-positive group (26.7 months, and 54.7 months, respectively), indicating a negative impact of biliary contamination on long-term outcomes (Figure 3).

The results of the multivariable Cox proportional hazards regression model are highly significant (Chi-square = 17.653, *p* < 0.001). Both positive bacterial culture and cancer disease staging were found to be statistically significant predictors of reduced OS (HR = 1.95, *p* = 0.019, and HR = 2.29, *p* < 0.001, respectively). Importantly, the interaction term between culture status and staging (positive culture × staging) was not statistically significant (HR = 0.60, *p* = 0.463), indicating that the prognostic effect of bacterial colonization does not differ depending on the pancreatic cancer stage (Table 5).

## 4. Discussion

Despite many advances in pancreatic surgery, there remains a lack of an effective strategy to reliably reduce or prevent POPF after a PD. Accurate risk prediction is essential not only for perioperative planning but for individualized patient consent. Although several risk scores have been developed—incorporating factors such as pancreatic duct diameter, gland texture, bilirubin levels, BMI, tumor type, and sex—none have achieved universal validation or consistently high predictive values [21,22,23]. The findings of our study support the hypothesis that bacterial colonization of bile may be associated with the development of POPF and a shortened OS after a PD.

Our findings revealed a borderline statistical association between bacteriobilia and the development of POPF grade B, with a substantial effect size observed in a univariate analysis (OR 5.11, *p* = 0.088). A subsequent multivariate analysis confirmed that bacteriobilia is a significant risk factor for both POPF grade B and clinically relevant POPF (OR 8.04, *p* = 0.048, and OR 5.50, *p* = 0.034, respectively). Notably, our observations are consistent with previous studies that have reported similar, significant associations [13,14,24].

A meta-analysis of eight studies conducted by Filson et al. did not find a statistically significant association between positive bile cultures and the development of POPF [25]. The Filson study included highly heterogeneous patient populations, frequently combining different tumor types and surgical indications, which may have masked associations detectable in more well-defined cohorts. By contrast, our study focused exclusively on a homogenous group of patients undergoing a PD for PDAC, thereby minimizing selection bias and allowing for a more focused assessment of microbial influence. Moreover, the meta-analysis combined all POPF events into a single outcome without distinguishing the fistula grade, potentially attenuating the observed effect of microbiological factors. Although individual studies within the analysis, including those by Ohgi et al. and Pretzsch et al., identified associations between specific pathogens and clinically relevant POPF, these associations may have been obscured by aggregation across heterogeneous study populations [13,14].

Similarly, in our study, BRM in bile were associated with higher rates of POPF grade C in both the univariable (OR 4.97; *p* = 0.026) and the multivariable analyses (OR 6.17; *p* = 0.047), supporting the hypothesis that a presence of BRM in bile may impair anastomotic healing and promote the progression to severe pancreatic fistula.

Our Kaplan–Meier survival analysis revealed a significantly shorter OS in patients with positive intraoperative bile cultures compared to those without (log-rank *p* = 0.009). Notably, the median OS was nearly twice shorter in the bile-positive group (26.7 months vs. 54.7 months), suggesting that biliary colonization may unfavorably affect long-term outcomes. A multivariable Cox regression analysis was conducted to determine whether a positive bile culture serves as an independent predictor of OS. The results demonstrated that both bacterial bile colonization and cancer advancement were independently associated with reduced survival. Notably, the interaction between culture status and cancer stage was not statistically significant (*p* = 0.463), indicating that the effect of bacterial colonization on survival did not vary according to disease stage.

Our findings are concordant with both preclinical and clinical data demonstrating that bacterial contamination alters the antitumor activity of bile; sterile bile reduced pancreatic cancer cell viability in both in vitro and in vivo models, whereas contaminated bile exhibited attenuated or even opposing effects [26,27,28]. A recent study by Jiang et al. demonstrated that alterations in bile microbial composition significantly correlated with progression-free survival in pancreatic cancer patients [29]. Hence, the microbiome may contribute to chemotherapy resistance through several pathways. Certain bacterial species are capable of enzymatically converting prodrugs into their active metabolites or, conversely, inactivating chemotherapeutic agents, thereby diminishing their cytotoxic effects on cancer cells. Additionally, microbiota can significantly influence the pharmacokinetics and pharmacodynamics of anticancer drugs by modulating their metabolism, bioavailability, and elimination [28,30,31,32].

Geller et al. demonstrated in a murine model that microbiota contributes to chemoresistance by administering gemcitabine with or without ciprofloxacin [31]. Antibiotic-treated mice showed effective bacterial depletion and a significantly enhanced antitumor response, while the controls exhibited rapid tumor progression (*p* < 0.001) [31]. Subsequent studies have corroborated these observations, demonstrating that the microbiome may also influence the therapeutic efficacy of other chemotherapeutic agents, including oxaliplatin and paclitaxel [30]. As shown in Figure 2, bile cultures revealed a broad bacterial spectrum, predominantly *Enterobacterales* and *Enterococcus* spp., with 16.04% of colonized patients harboring at least one BRM. The microbiota profile—dominated by *Enterococcus faecalis* (18.39%), *Escherichia coli* (14.35%), and *Klebsiella pneumoniae* (12.11%)—was consistent with previous studies [12,14,24,25,33,34]. These pathogens have been associated with both severe postoperative complications and diminished oncological outcomes. Notably, *Klebsiella pneumoniae* has been linked to reduced gemcitabine efficacy [28], while *Enterococcus faecalis* has been implicated in higher perioperative mortality [35]. Our findings align with those of Hoffmann et al., who reported that similar biliary pathogens were significant risk factors for clinically relevant POPF, further supporting their potential role in adverse postoperative outcomes [36].

This high prevalence of bacteria in bile, particularly BRM, suggests a need to reevaluate standard perioperative antibiotic strategies. In selected high-risk patients—particularly those with positive intraoperative bile cultures or clinical predictors of bacterial biliary colonization—early and broader-spectrum antimicrobial prophylaxis or even perioperative antibiotic treatment may be warranted. Our study, along with prior reports, observed an association between preoperative ERCP and bacteriobilia, with bile colonization frequently identified in patients undergoing ERCP, and those patients might be good candidates for different antibiotic administration before a PD [12,24,33,34]. However, the indications for ECPW (cholangitis/elevated bilirubin levels) and their association with possible bile contamination were not analyzed in this study. This issue is the subject of ongoing research, which is being prepared for publication.

Our findings suggest that bile colonization could contribute to anastomotic complications and shorten the long term survival. Therefore, in line with the recommendations of Krüger et al. and Stein-Thoeringer et al. [33,35], we suggest that alternative perioperative antibiotic prophylaxis regimens—such as piperacillin–tazobactam or, as indicated by recently published data, amoxicillin–clavulanate combined with gentamicin [37]—may represent a rational and clinically relevant strategy and should be considered in patients undergoing a pancreatoduodenectomy.

This study has several important limitations that should be acknowledged. First, its retrospective design and single-center setting limit the ability to draw causal inferences, particularly with respect to survival outcomes. As such, the findings should be interpreted as hypothesis-generating rather than definitive evidence of causality. In addition, the impact of adjuvant chemotherapy on survival was not analyzed in the present study. All patients were managed according to an intention-to-treat strategy, and postoperative antibiotic therapy was initiated or adjusted based on clinical status and microbiological findings, particularly in cases of suspected infection or sepsis. However, data regarding the timing, type, and completion of adjuvant chemotherapy were not uniformly available and could not be reliably assessed.

Finally, the limited number of events for some outcomes may have reduced the precision of the effect estimates, as reflected by wide confidence intervals in selected analyses. Despite these limitations, the findings offer clinically relevant insights and help define the priorities for future prospective and interventional studies.

## 5. Conclusions

Our findings indicate that bacterial colonization of bile may contribute to early postoperative complications, including POPF, and that bacteriobilia may serve as a potential prognostic factor for shortened survival following a PD. The presented results support a consideration of microbiota-guided perioperative approaches, including preoperative biliary decontamination as a potential strategy to improve short- and long-term outcomes in patients undergoing pancreatic resection. Further prospective studies in larger cohorts are required to validate these associations and to clarify whether modulation of the biliary microbiome has a role as an adjunct in the surgical management of pancreatic cancer.

## Figures and Tables

**Table 1:** Characteristics of the study population, incidence of bile colonization, and postoperative pancreatic fistula (POPF).

Variable	Mean ± SD/Total (*n* = 138) (%)	Range or Categories
Age (years)	66.3 ± 9.8	37–89
BMI (kg/m^2^)	25.2 ± 4.6	16.4–42.2
Male sex	72 (52.2%)	Male/Female
Preoperative diabetes mellitus	34 (24.6%)	
American Society of Anesthesiologists (ASA) classification:		
I II III	18 (13.1%) 87 (63.0%) 33 (23.9%)	
Preoperative Endoscopic Retrograde Cholangiopancreatography (ERCP)	101 (73.2%)	
Positive bile culture (Bacteriobilia)	106 (76.8%)	
Positive bile culture in patients after preoperative ERCP	94 (93.1% ^a^)	
Bacteria with resistance mechanisms (BRM)	17 (12.3%)	
POPF clinically relevant:	24 (17.4%)	
POPF grade B	16 (11.6%)	
POPF grade C	8 (5.8%)	

^a^ Percentage of all patients after ERCP (*n* = 101).

**Table 2:** Characteristics of bacteria with resistance mechanisms.

Bacterial Species	Resistance Mechanism	Total (*n* = 23) (%)
*Enterococcus casseliflavus*	VRE	2 (8.7%)
*Enterococcus gallinarum*	VRE	1 (4.3%)
*Klebsiella pneumoniae*	ESBL + NDM	3 (13.0%)
*Klebsiella pneumoniae*	ESBL	7 (30.4%)
*Klebsiella oxytoca*	ESBL	1 (4.3%)
*Escherichia coli*	ESBL	7 (30.4%)
*Enterobacter aerogenes*	ESBL	1 (4.3%)
*Enterobacter cloacae*	ESBL	1 (4.3%)

VRE—vancomycin-resistant enterococcus; ESBL—extended-spectrum beta-lactamases; NDM—New Delhi metallo-β-lactamase.

**Table 3:** Association between bile colonization and the occurrence of different types of postoperative pancreatic fistula (POPF).

POPF Grade B		
Bacteria status:	OR with 95% CI	*p*-value
negative	1.00	
positive	5.11 (0.65–40.30)	0.088
Bacteria resistance:		
no BRM	1.00	
BRM	1.02 (0.21–4.93)	0.981
**POPF Grade C**		
Bacteria status:	OR with 95% CI	*p*-value
negative	1.00	
positive	0.48 (0.11–2.12)	0.323
Bacteria resistance:		
no BRM	1.00	
BRM	OR 4.97 (1.071–23.07)	0.026 *

* indicates statistical significance.

**Table 4:** Multivariable logistic regression analysis of microbiological risk factors for POPF, adjusted for age, sex, BMI, ASA physical status, and type of pancreatic duct anastomosis technique. The table presents OR, 95% confidence intervals (95% CI), and *p*-value.

	POPF Grade B	POPF Grade C	POPF Clinically Relevant
Bacteriobilia	OR 8.04 (0.98–66.18); *p* = 0.048 *	OR 1.61 (0.16–16.48); *p* = 0.686	OR 5.50 (1.14–26.6); *p* = 0.034 *
BRM	OR 0.63 (0.12–3.33); *p* = 0.590	OR 6.17 (1.02–45.61); *p* = 0.047 *	OR 1.66 (0.45–6.02); *p* = 0.444
Age	OR 0.997 (0.95–1.05); *p* = 0.942	OR 0.95 (0.87–1.05); *p* = 0.316	OR 0.985 (0.94–1.04); *p* = 0.558
Sex (male)	OR 1.58 (0.53–4.71); *p* = 0.417	OR 1.67 (0.29–10.02); *p* = 0.560	OR 1.72 (0.65–4.56); *p* = 0.278
BMI	OR 1.05 (0.94–1.17); *p* = 0.378	OR 1.05 (0.90–1.23); *p* = 0.525	OR 1.06 (0.96–1.17); *p* = 0.257
ASA	OR 0.39 (0.11–1.41); *p* = 0.151	OR 0.55 (0.09–3.32); *p* = 0.515	OR 0.37 (0.12–1.15); *p* = 0.087
Pancreatic anastomosis technique (duct to mucosa)	OR 0.94 (0.25–3.46); *p* = 0.921	OR 0.59 (0.12–3.07); *p* = 0.533	OR 0.75 (0.25–2.29); *p* = 0.619

* indicates statistical significance.

**Table 5:** Summary of Cox regression results including interaction term.

Variable	HR (95% CI)	*p*-Value
Positive culture	1.95 (1.12–3.41)	0.019 *
Staging	2.29 (1.40–3.75)	0.001 *
Positive culture × Staging (interaction)	0.60 (0.15–2.37)	0.463

Chi-square = 17.653, *p* < 0.001, * indicates statistical significance.

## Data Availability

Data are available upon request.

## Abbreviations

The following abbreviations are used in this manuscript:

AMR	Antimicrobial Resistance
BRM	Bacteria with Resistance Mechanisms
ERCP	Endoscopic Retrograde Cholangiopancreatography
PBD	Preoperative Biliary Drainage
PC	Pancreatic Cancer
PD	Pancreatoduodenectomy
PDAC	Pancreatic Ductal Adenocarcinoma
POPF	Postoperative Pancreatic Fistula
